# Co-cultivation of the strictly anaerobic methanogen *Methanosarcina barkeri* with aerobic methanotrophs in an oxygen-limited membrane bioreactor

**DOI:** 10.1007/s00253-018-9038-x

**Published:** 2018-05-03

**Authors:** Michiel H. in ’t Zandt, Tijs J. M. van den Bosch, Ruud Rijkers, Maartje A. H. J. van Kessel, Mike S. M. Jetten, Cornelia U. Welte

**Affiliations:** 1 Department of Microbiology, Institute for Water and Wetland Research, Radboud University, Heyendaalseweg 135, 6525 AJ Nijmegen, The Netherlands; 2 Netherlands Earth System Science Center, Utrecht University, Heidelberglaan 2, 3584 CS Utrecht, The Netherlands; 3 Soehngen Institute of Anaerobic Microbiology, Radboud University, Heyendaalseweg 135, 6525 AJ Nijmegen, The Netherlands

**Keywords:** Co-culture, Methane cycle, Aggregation, Methanogens, Methanotrophs

## Abstract

Wetlands contribute to 30% of global methane emissions due to an imbalance between microbial methane production and consumption. Methanogenesis and methanotrophy have mainly been studied separately, and little is known about their potential interactions in aquatic environments. To mimic the interaction between methane producers and oxidizers in the environment, we co-cultivated the methanogenic archaeon *Methanosarcina barkeri* with aerobic *Methylocystaceae* methanotrophs in an oxygen-limited bioreactor using acetate as methanogenic substrate. Methane, acetate, dissolved oxygen, available nitrogen, pH, temperature, and cell density were monitored to follow system stability and activity. Stable reactor operation was achieved for two consecutive periods of 2 months. Fluorescence in situ hybridization micrographs indicated close association between both groups of microorganisms. This association suggests that the methanotrophs profit from direct access to the methane that is produced from acetate, while methanogens are protected by the concomitant oxygen consumption of the methanotrophs. This proof of principle study can be used to set up systems to study their responses to environmental changes.

**Electronic supplementary material:**

The online version of this article (10.1007/s00253-018-9038-x) contains supplementary material, which is available to authorized users.

## Introduction

Wetlands are the biggest natural methane source and contribute 30% to global methane emissions (167 Tg CH_4_/year) (Saunois et al. 2016). Methane is an important greenhouse gas (GHG) with a 34-fold higher warming potential than CO_2_ (Myhre et al. 2013). Eighteen percent of the total global greenhouse effect is currently attributed to methane (Prather et al. 2012; Myhre et al. 2013). In wetlands, methanogenic archaea carry out the final reaction in the anaerobic degradation of organic matter resulting in methane production. Wetland methane emissions are mitigated by the activity of both anaerobic methanotrophic bacteria and archaea (Raghoebarsing et al. 2006; Ettwig et al. 2010; Haroon et al. 2013) and aerobic methanotrophic bacteria (reviewed by Hanson and Hanson 1996). Aerobic methanotrophy has been estimated to be the most significant methane oxidation pathway in cold ecosystems (Mackelprang et al. 2011; Barbier et al. 2012; Knoblauch et al. 2013), although anaerobic methane oxidizers have also been detected in cold freshwater and peatland ecosystems (Smemo and Yavitt 2011; Gupta et al. 2013; Kao-Kniffin et al. 2015). Early studies on lake and peatland systems indicated that aerobic methanotrophs have the potential to oxidize up to 95% of the methane that is produced (Yavitt et al. 1988; Frenzel et al. 1990). Spatial coexistence has been observed in, for example, cooperation of nitrogen cycle microorganisms (Sliekers et al. 2002; Yang et al. 2012). Several studies implied that this coexistence in seemingly anoxic environments is probably enabled due to high oxygen consumption rates (Oswald et al. 2016; Martinez-Cruz et al. 2017). In addition, aerobic methanotrophs are tolerant to long periods of anoxic conditions (Roslev and King 1994).

The interactions between methanogens and aerobic methanotrophs that may strongly control the GHG fluxes of cold wetland ecosystems remain poorly understood (Bridgham et al. 2013). Only few studies on methane fluxes in oxic-anoxic systems have been done so far (Gerritse and Gottschal 1993; Shen et al. 1996; Miguez et al. 1999). Shen et al. designed a bioreactor with an aerobic-anaerobic interface using a granular sludge bed that allowed for sufficient methanogenic activity to support growth of the aerobic methanotroph *Methylosinus sporium* (Shen et al. 1996). However, this system did not employ axenic cultures and observations showed gradual reduction of *M. sporium*, indicating competition for oxygen with facultative anaerobic bacteria. Similarly, Miguez et al. used an upflow anaerobic sludge blanket (UASB) reactor to co-cultivate complex methanogenic cultures with the aerobic methanotrophs *Methylosinus trichosporium* and *M. sporium* (Miguez et al. 1999). Gerritse and Gottschal were the first to set up defined co-cultures with *Methanosarcina barkeri* and *Methanobacterium formicicum* together with aerobic methanotrophic *Methylocystis* sp. (Gerritse and Gottschal 1993). However, in-depth data on species interactions are lacking. Here, we established a co-culture of the methanogen *M. barkeri* and aerobic *Methylocystaceae* methanotrophs in a membrane bioreactor to generate a method to study interspecies interactions between methane cycle microorganisms. Under oxygen-limited conditions, a stable co-culture was monitored over time and several key parameters were determined.

## Materials and methods

### Strains

*M. barkeri* DSM 800 and *Methylosinus sporium* DSM 17706 strains were ordered from the DSMZ (Leibniz Institute DSMZ-German Collection of Microorganisms and Cell Cultures, Braunschweig, Germany) as actively growing cultures. *M. sporium* was chosen for high substrate affinity (Murrell et al. 2000) and co-occurrence in methanogenic-methanotrophic cultures (Shen et al. 1996; Miguez et al. 1999). Genomic analysis of the *M. sporium* culture indicated presence of a second strain of another *Methylocystaceae* species related to *Methylocystis rosea,* as described in detail in the “Results” section. *M. barkeri* was chosen for the oxygen-limited reactor set-up due to its relative high oxygen tolerance (up to several hours under atmospheric oxygen levels) and wide substrate range (Kiener and Leisinger 1983; Brioukhanov et al. 2000; Maeder et al. 2006). Furthermore, its reference genome is available (PRJNA230939). *M. barkeri* can perform acetoclastic methanogenesis via acetate dismutation to methane and CO_2_. In addition, *M. barkeri* can be grown on mineral media, a prerequisite for this co-cultivation study (Maestrojuan and Boone 1991). *M. sporium* was pre-grown on general medium as described at “Reactor set-up and co-cultivation.” *M. barkeri* was pre-grown on methanogen medium (1.7 mM KH_2_PO_4_, 0.9 mM NH_4_Cl, 0.2 mM MgSO_4_·7H_2_O, 0.2 mM CaCl_2_·2H_2_O, 0.5 mM NaCl, 0.7 μM FeSO_4_·7H_2_O, 0.25 g/L Tryptone, 0.25 g/L yeast extract, 1 mL 1000× trace elements SL-6 including 81.5 μM CeCl·7H_2_O (DSMZ, Braunschweig, Germany), and 1 mL 1000× vitamin solution (DSMZ, Braunschweig, Germany) with 100 mM acetate and using 0.001% resazurin (*w*/*v*) as redox indicator). pH was adjusted to 8.5 with sodium hydroxide, and bottles were made anoxic with a triplicate + 1 bar overpressure gas followed by a 15 min vacuum cycle using a 9:1 Argon/CO_2_ gas mixture resulting in a pH of around 7.0 after autoclaving.

### Reactor set-up and co-cultivation

#### Reactor set-up

**Fig. 1:**
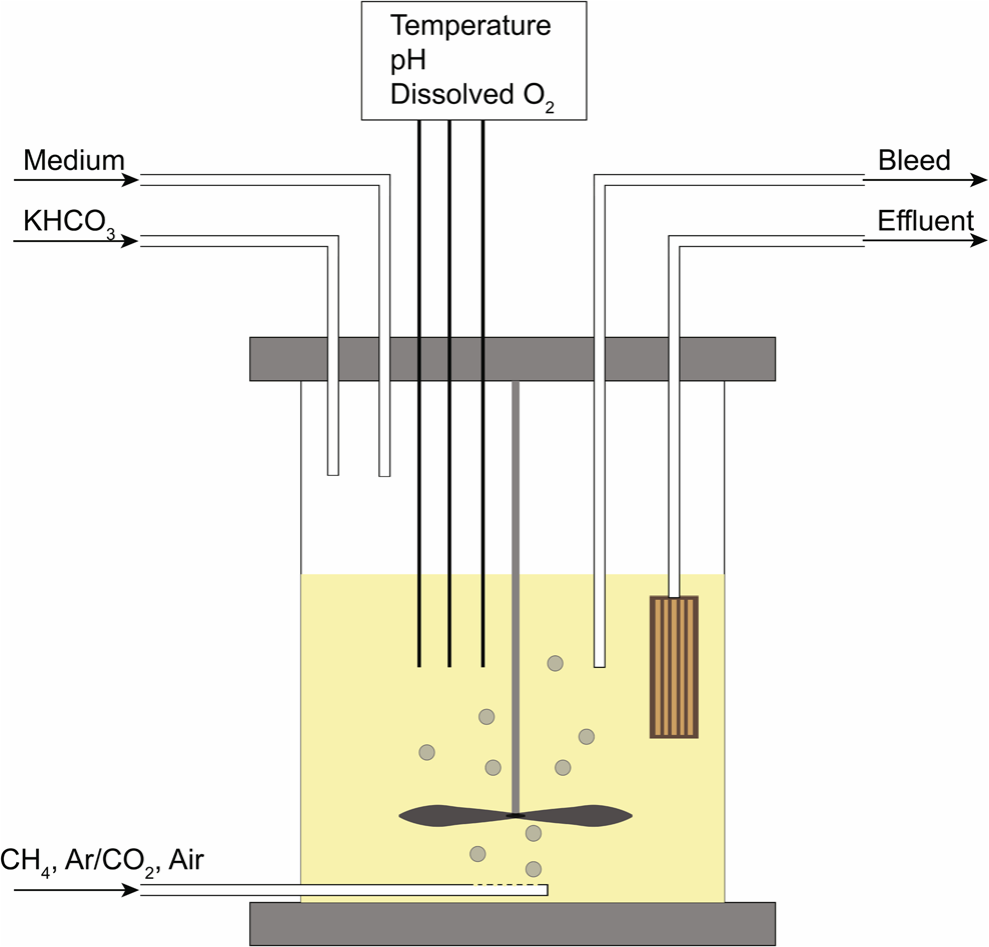
Set-up of the 2 L membrane bioreactor (MBR) with a constant volume of 1.5 L. The inflow gas was a mixture of CH_4_, Argon and CO_2_, and compressed air of which the ratios could be modified. Medium and buffer (KHCO_3_) inflows and bleed and effluent outflows were controlled by calibrated medium pumps. Temperature, pH and dissolved O_2_ were constantly monitored using in-liquid probes. Contents were mixed at 150 rpm with a stainless steel rotor blade

A 2 L membrane bioreactor (MBR, see Yoon (2016) for details) with an operational volume of 1.5 L, and a settling and bleed cycle to control growth rate, and to select for aggregates, was designed for the co-culturing of methanogens and aerobic methanotrophs (Fig. 1). A general medium was devised that allowed growth of both methanogens and methanotrophs (1.0 mM MgSO_4_·7H_2_O, 0.23 mM CaCl·2H_2_O, 1.7 mM KH_2_PO_4_, 5.1 mM NaCl, 7.2 μM FeSO_4_·7H_2_O, 3.7 mM NH_4_Cl, 26.2 mM CH_3_COOH, 0.25 g/L Tryptone, 0.25 g/L yeast extract, 1 mL 1000× trace elements SL-6 including 81.5 μM CeCl·7H_2_O (DSMZ, Braunschweig, Germany), and 1 mL 1000× vitamin solution (DSMZ, Braunschweig, Germany)). pH was adjusted to 7.0. At *t* = 111 days, Tryptone and yeast extract were removed from the medium to reduce risk of contamination in the bioreactor. Growth tests on Tryptone and yeast extract free medium confirmed that growth of both methanogens and methanotrophs was possible (data not shown). The total medium flow supply was set to 0.5 L/day, and the volume was kept constant at 1.5 L using a level-controlled effluent pump. The gas flow rate was set to 5 mL/min. Initial gas mixture composition was set to 1.51 mL Argon/CO_2_ (9:1) mixture, 2.38 mL air, and 0.75 mL methane. After 15 weeks of co-cultivation, the gas inflow was increased to 10 mL/min to reduce the risk of air diffusion into the reactor. Simultaneously, the methane inflow percentage was reduced based on the prevailing methane consumption data. The mixture contained 6.77 mL argon/CO_2_, 2.86 mL air, and 0.37 mL methane per minute. O_2_ and pH were monitored using AppliSens probes (AppliSens Z001023551 and Applisens Z010023520, Applikon Biotechnology B.V., Delft, The Netherlands). The pH was controlled at 7.0 ± 0.1 using a probe-linked KHCO_3_ pump. The MBR was continuously mixed at 150 rpm using a stainless steel rotor blade controlled by an Applikon stirrer controller (Applikon stirrer controller P100, Applikon Biotechnology B.V., Delft, The Netherlands). The system was operated at room temperature (± 20 °C).

#### Co-cultivation

The methanotroph-only culture was grown to OD600 of 0.7 under oxygen limitation, which created anoxic conditions in the liquid. After at least a week of anoxia (O_2_ detection limit 3 μM), the reactor was inoculated with *M. barkeri*. Fourteen days after inoculation with *M. barkeri*, a bleed cycle was introduced to select for methanogen-methanotroph aggregates and to remove excess methanotrophic biomass. The bleed cycle was set to 30 min pre-settling (no stirring) to minimize loss of aggregates followed by removal of 50-mL reactor fluid from the upper layer in 20 min. Triplicate samples were taken every 3–4 days. Cells and supernatant were taken aseptically from the MBR and separated by centrifugation for 10 min at maximum speed (20,348×*g*) and used for protein determination, FISH microscopy, and activity tests.

### Monitoring of growth and substrate metabolism

#### OD600 and protein assay

After sterilization, the bioreactor was inoculated with *M. sporium*. Batch culture and reactor optical density were measured on a Bio-Rad SmartSpec™ 3000 spectrophotometer with the pre-set OD600 method (Bio-Rad Laboratories, Veenendaal, The Netherlands) using 1.6-mL semi-micro polystyrene cuvettes (Sarstedt AG & Co. KG, Nümbrecht, Germany). For analysis of total protein contents, duplicate 1.5 mL reactor liquid pellets were resuspended in 0.3 mL 3 M NaOH and boiled for 10 min at 95 °C. Samples were cooled down to RT, neutralized with 0.3 mL 3 M HCl, and centrifuged for 1 min at maximum speed. Ten microliters supernatant was loaded onto a polystyrene flat-based 96-well microtest plate (Sarstedt AG & Co. KG, Nümbrecht, Germany). To each well, 200 μl working solution (50 parts of Pierce BCA Protein Assay Kit, reagent A (article no 23223) and 1 part of 4% cupric sulfate in Milli-Q water was added and mixed by pipetting. Samples were incubated for 30 min at 60 °C. After cooling down to room temperature, the absorbance was measured at 562 nm on a SpectraMax 190 Microplate Reader (Molecular Devices, Sunnyvale CA, USA), and data were analyzed using SoftMax Pro 6.4 (Molecular Devices, Sunnyvale CA, USA). Values were compared to a standard curve with bovine serum albumin (BSA) in Milli-Q. OD600 values and total protein analysis achieved similar results (Supplementary Information Fig. S1).

#### Methane and oxygen

For all gas determinations, 50 μl gas samples were withdrawn with a gas-tight glass syringe (Hamilton, Reno NE, USA). Methane in- and outflow concentrations were measured daily using a HP5890a gas chromatograph (Hewlett Packard 5890a, Agilent Technologies, Santa Clara CA, US) equipped with a Porapaq Q 100/120 mesh and a thermal conductivity detector (TCD) using N_2_ as carrier gas (Sigma-Aldrich, Saint Louis MI, USA). Data were analyzed using GC ChemStation Rev. A.10.02 (Agilent Technologies, Santa Clara CA, USA). Oxygen concentrations were determined with the same gas sampling and volume on an Agilent 6890 series gas chromatograph coupled to a mass spectrometer (Agilent Technologies, Santa Clara CA, USA) equipped with a Porapaq Q column heated at 80 °C with helium as carrier gas. Data were analyzed with the MSD ChemStation F01.01.2317 (Agilent Technologies, Santa Clara, CA, USA). All gas measurements were performed in duplicate.

#### Acetate

Acetate concentrations were determined according to the protocol described by Kage and co-workers with the following modifications: An internal standard (IS) was prepared by dissolving 0.1 mM methylstearate (MS) in n-hexane (Kage et al. 2004). For each reaction, 40 μl supernatant sample, 40 μl 0.5 M phosphate-buffered saline (PBS 65 mM NaCl, 5 mM phosphate buffer pH 7.4 (80% Na_2_HPO_4_ and 20% NaH_2_PO_4_)), and 200 μl pentafluorobenzyl bromide (PFBBr) in acetone at a concentration of 100 mM were mixed and incubated for 1 h at 60 °C. Acetate standard solutions were prepared according to Kage et al. (2004) using sodium acetate in Milli-Q water with a concentration range from 0 to 10 mM. Four hundred microliters of the 0.1 mM MS solution in n-hexane was added, and samples were vortexed for 1 min at RT and centrifuged for 2 min at maximum speed. One hundred microliters aliquots were divided into 12 × 32 mm clear Cronus crimp vials capped with rubber/PTFE snap caps (SMI-LabHut Ltd., Maisemore, UK); 0.1 mL 15 mm tip clear glass inserts were used to reduce the required sample volume (VWR International BV, Amsterdam, The Netherlands). Pure n-hexane was added to the vials to avoid excess sample evaporation. All samples were injected five times on a JEOL AccuTOF-GCv JMS-100GCv (JEOL Ltd., Akishima, Tokyo, Japan).

#### Ammonium and nitrite

Ammonium concentrations were measured using 50 μl supernatant sample and 750 μl OPA Reagent (0.54% (*w*/*v*) ortho-phthaldialdehyde, 0.05% (*v*/*v*) β-mercaptanol and 10% (*v*/*v*) ethanol in 400 mM potassium phosphate buffer (pH 7.3)). Samples were vortexed shortly and incubated at room temperature (RT) for 20 min. Absorbance was measured at 420 nm and values were compared to a calibration curve with ammonium chloride in Milli-Q. Nitrite concentrations were measured using 100 μl sample, 100 μl nitrite reagent (1% (*w*/*v*) sulfanilic acid in 1 M HCl and 100 μl 0.1% (*w*/*v*) naphtylethylene diaminedihydrochloride in water). Samples were mixed by pipetting and incubated for 20 min at RT. Standard curves were prepared by using a dilution series of sodium nitrite in Milli-Q. Absorbance was measured at 540 nm. Both assays were performed in triplicate in a 96-well plate set-up as described for the protein assay.

### Fluorescence in situ hybridization (FISH) microscopy

FISH microscopy samples were taken weekly and prepared in duplicate according to the FISH protocol as described by Amann et al. (1990) using a hybridization buffer with 35% (*v*/*v*) formamide. *M. sporium* was targeted using bacterial EUB mix probes (5′-GCT GCC TCC CGT AGG AGT-3′; 5′-GCA GCC ACC CGT AGG TGT-3′; 5′-GCA GCC TTC CGT AGA AGT-3′) (Daims et al. 1999). *M. barkeri* was targeted by a combination of ARCH-0890 probe (5′-GTG CTC CCC CGC CAA TTC CT-3′) targeting archaea (Stahl and Amann 1991) and MSMX-0860 probe (5′-GGC TCG CTT CAC CGC TTC CCT-3′) targeting *Methanosarcinaceaea* (Raskin et al. 1994).

### Genome sequencing and data analysis of methanotrophic culture

DNA was extracted from 20 mL pelleted methanotrophic culture grown to an OD600 of ~ 1. DNA was extracted using the cetyltrimethylammoniumbromide (CTAB) extraction buffer protocol as described by Zhou et al. (1996). DNA library preparation and sequencing was performed by BaseClear on an Illumina HiSeq2500 platform using the Illumina paired end protocol (BaseClear B.V., Leiden, The Netherlands). Quality-trimming, adapter removal, and contaminant-filtering of Illumina HiSeq paired-end sequencing reads was performed using BBDUK (BBTOOLS version 37.76) (Bushnell 2014). Trimmed reads were assembled using metaSPAdes v3.11.1 (Nurk et al. 2017) at default settings. MetaSPAdes iteratively assembled the metagenome using k-mer size 21, 33, 55, 77, 99, and 127. Reads were mapped back to the metagenome using Burrows-Wheeler Aligner 0.7.17 (BWA) (Li and Durbin 2018), employing the “mem” algorithm. The sequence mapping files were handled and converted as needed using SAMtools 1.6 (Li et al. 2018). Metagenome binning was performed for contigs greater than 1500 bp using five binning algorithms: BinSanity v0.2.6.1 (Graham et al. 2017), COCACOLA (Lu et al. 2018), CONCOCT (Alneberg et al. 2014), MaxBin 2.0 2.2.4 (Wu et al. 2015), and MetaBAT 2 2.12.1 (Kang et al. 2015). The bin sets from each algorithm were supplied to DAS Tool 1.0 (Sieber et al. 2017) for consensus binning to obtain the final optimized bins. The quality of the generated bins was assessed through single-copy marker gene analysis using CheckM 1.0.7 (Parks et al. 2015). Genomes were annotated with Prokka 1.12 (Seemann 2014) using the NCBI Reference Sequence Database (RefSeq) release 85 (Pruitt et al. 2018). Predicted protein sequences were submitted to the KEGG Automatic Annotation Server (KAAS—last update April 3, 2015) (Moriya et al. 2007) for pathway analyses. Genome annotations were examined using the Artemis genome browser release 16.0.0 (Rutherford et al. 2000). For 16S rRNA gene analysis, raw Illumina HiSeq reads were mapped against the SILVA SSU non-redundant database version 128 and de novo assembled as described in in ’t Zandt et al. (2017).

### Nucleotide sequence accession numbers

All sequencing data were submitted to the GenBank databases under BioProject PRJNA434352. The genome bins were submitted as genome data under BioSample accession number SAMN08554708 and SAMN08554709.

## Results

### Start-up of the oxygen-limited bioreactor

**Fig. 2:**
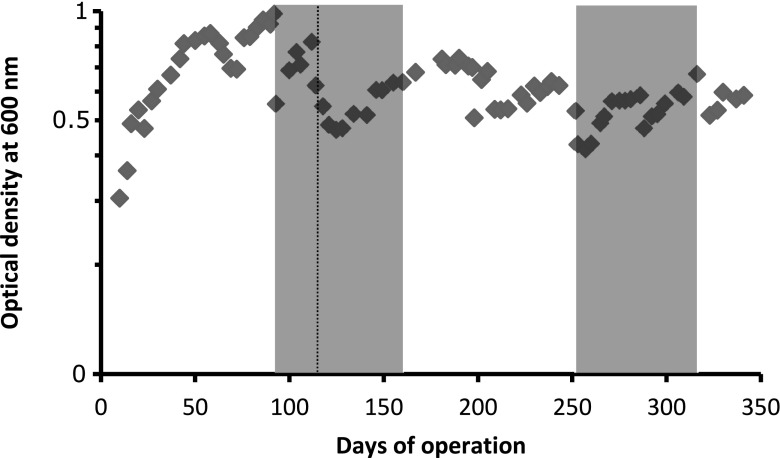
Optical density of biomass during operation of the membrane bioreactor. Gray areas indicate co-cultivation periods. At *t* = 92, methanogens were added. The first co-cultivation lasted until *t* = 155 days. At *t* = 252, a second batch of *M. barkeri* was added. The second co-cultivation lasted until *t* = 317. The dashed line indicates installation of the bleed at *t* = 113

A membrane bioreactor was inoculated with aerobic methanotrophs and fed with a gas mixture containing methane and oxygen. A stable co-culture of anaerobic methanogens and aerobic methanotrophs could be obtained for over 2 months. Optical density (OD) was followed over time (Fig. 2). The initial phase with only aerobic methanotrophs indicated a rapid growth based on an OD600 increase from 0.3 to 0.85 within 55 days. The methanotrophs were put under oxygen limitation, and after the dissolved oxygen levels dropped below the detection limit (≤ 3 μM), the OD600 was diluted to 0.5 and the reactor was inoculated on *t* = 92 days with the methanogenic archaeon *M. barkeri*. Oxygen levels remained below the detection limit for the entire experiment. At *t* = 105 days, methane inflow was reduced to 1.25 mL/min to restrict methanotrophic growth. Co-culture OD values increased to almost 1 within 19 days. At *t* = 113 days, additional biomass was removed and a bleed was installed to remove 1/30th of the reactor volume per day to target an OD600 of around 0.5. From *t* = 113 days to the end of reactor operation at *t* = 337 days OD600 values ranged from 0.4 to 0.7.

### Reducing methane flux results in higher reactor stability

**Fig. 3:**
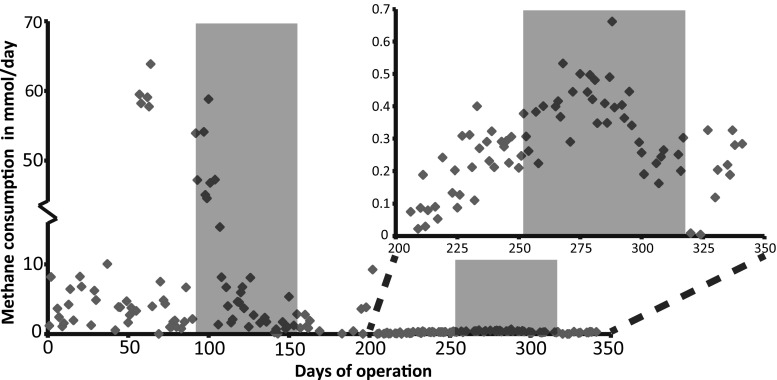
Total methane consumption in mmol/day as measured by reactor inflow and outflow gas methane concentrations. Gray areas indicate co-cultivation periods. At *t* = 92, methanogens were added. The first co-cultivation lasted until *t* = 155 days. At *t* = 252, a second batch of *M. barkeri* was added. The second co-cultivation lasted until *t* = 317. Right top graph shows zoom-in from *t* = 200 to *t* = 350 days

Inflow and outflow methane concentrations were measured to follow methanotrophic activity (Fig. 3). During pre-cultivation with methanotrophs (*t* = 0 to 92 days), methane consumption was highly variable as indicated by the deviation in the data. Directly after the inoculation with *M. barkeri* at t = 92 days, the total methane consumption rates increased about tenfold from days 92 to 104 with an average of 41.4 ± 0.8 (standard error, SE) mmol/day, probably due to removal of excess (inactive) biomass prior to inoculation. A similar observation was made between *t* = 55 and 65 days after excess biomass was removed. Methane consumption rates increased to an average of 43.5 ± 1.4 (SE) mmol/day. On *t* = 205 days, methane influx was lowered to 0.62 mL/min to reduce methanotrophic growth and to make the system more dependent on internally produced methane. After second addition of *M. barkeri* on *t* = 252 days, this resulted in the observation of up to ten times more methanogen-methanotroph aggregates.

Ammonium and nitrite concentrations were measured weekly within the co-cultivation periods from *t* = 83 to *t* = 315 days to monitor nitrogen availability, consumption, and nitrite toxicity risk (data not shown). Total available ammonium ranged between 2.3 and 4.7 mM with an average of 3.3 ± 0.5 (standard deviation, SD) mM.

### Monitoring showed constant availability and consumption of acetate

**Fig. 4:**
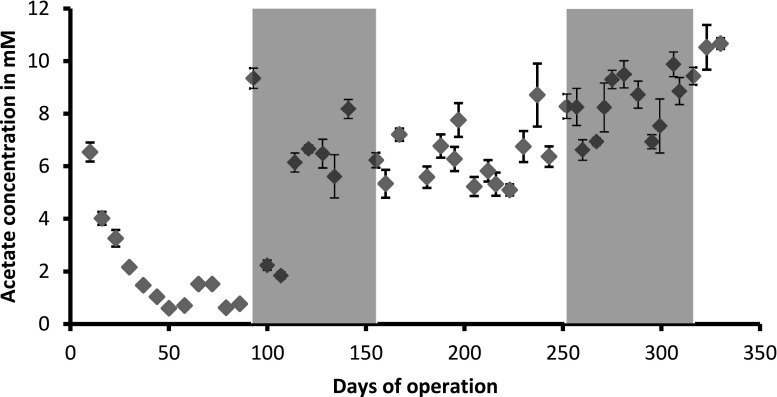
Weekly measurements of the acetate concentrations in the reactor liquid. Gray areas indicate co-cultivation periods. At *t* = 92, methanogens were added. The first co-cultivation lasted until *t* = 155 days. At *t* = 252, a second batch of *M. barkeri* was added. The second co-cultivation lasted until *t* = 317. Error bars indicate standard error of the mean, *n* = 5 technical replicate measurements on a JEOL AccuTOF-GCv

Five millimolars of acetate was added to the medium as methanogenic substrate. Acetate concentrations were monitored weekly (Fig. 4). These data indicated acetate consumption during the pre-cultivation period and thus suggested that the methanotrophic bacteria assimilated some of the acetate into biomass. *M. barkeri* Ks for acetate is 3–5 mM, and the threshold is 0.2–1.2 mM (Daniels 1993). Acetate concentrations were increased to 10 mM after 92 days to reduce acetate limitation risks for the methanogens. Further measurements indicated that acetate was not limiting during the entire co-cultivation periods.

### Fluorescence in situ hybridization showed clustering of methanogens and aerobic methanotrophs

**Fig. 5:**
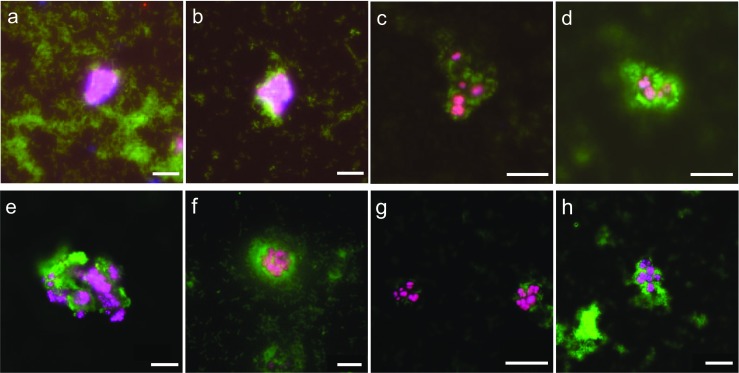
**a**–**d** Fluorescence in situ hybridization micrographs of the reactor biomass after 1 (**a**), 22 (**b**), 49 (**c**), and 63 days (**d**) of inoculation with *M. barkeri*. **e**–**h** Fluorescence in situ hybridization micrographs of the reactor biomass after 3 (**e**), 16 (**f**), 30 (**g**), and 65 days (**h**) of the second inoculation with *M. barkeri*. Aerobic methanotrophic bacteria are stained in green (EUB mix), and *M. barkeri* are stained in magenta (overlay of ARCH-0890 and MSMX-0860 in blue and red respectively). Scale bars represent 10 μm

Using fluorescence in situ hybridization (FISH) microscopy, the formation of aggregates containing both aerobic methanotrophs and anaerobic methanogens was detected over time (Fig. 5a–d). Abundance estimations from the FISH micrographs indicated a ratio of methanotrophs to methanogens of around 100:1, which was supported by the observed rapid growth of the methanotrophic population. First distinct aggregates were observed after 22 days of co-cultivation (Fig. 5b). The structure of the aggregates after 49 (*t* = 142) and 63 (*t* = 156) days of co-cultivation (Fig. 5c, d) showed a center or multiple centers of methanogen clusters (sarcina-shaped cell packages) surrounded by multiple cell layers of methanotrophs. This led to the hypothesis that both methanogens and aerobic methanotrophs profit from this specific spatial organization (Supplementary Information Fig. S2). Methanogenic capacity of the reactor contents was still retained when samples were batch cultured anaerobically with acetate as sole carbon source (data not shown).

### Lower methane/ammonium ratio induced nitrite toxicity event

After reducing the methane influx to 0.62 mL/min on *t* = 205 days, nitrite increased from background levels of 15.6 ± 10.8 (SD) μM to 78.6 ± 4.8 (SD), probably due to the non-specific co-metabolism of ammonia by methane monooxygenase (MMO) of the aerobic methanotrophs under methane-limiting conditions. The nitrite accumulation resulted in a decrease of *M. barkeri* cells as could be observed by FISH microscopy (data not shown). To minimize the risk of ammonium co-metabolism, ammonium concentration in the inflow medium was lowered from 3.7 to 1 mM. Growth experiments with axenic cultures of *M. barkeri* indicated that growth was not limited by ammonium concentrations down to 1 mM but was reduced when concentrations fell below 0.5 mM. Although the ammonium source is shared during co-cultivation, reactor liquid ammonium concentrations never dropped below 0.5 mM (data not shown). After system stabilization, a second inoculation with *M. barkeri* was performed (*t* = 252 days) to confirm the successful co-cultivation and to show repeated formation of interactions between methanogens and aerobic methanotrophs (Fig. 5e–h).

### Genome sequencing and analysis of aerobic methanotrophs

To confirm the identity of the methanotrophs, the culture was sequenced using Illumina HiSeq technology. Sequence analysis was performed using consensus binning, annotation, and de novo assembly based on a 16S rRNA gene identification approach. The culture appeared to contain two methanotrophic species. The first bin was assigned to *Methylosinus* (3.79 Mbp, GC-content 65.2%, N50 = 96,528 bp, 55 contigs) and had 100% completeness, 0.31% contamination, and no strain heterogeneity. The second bin was identified as *Methylocystis* (4.36 Mbp, GC-content 62.5%, N50 = 136,571 bp, 55 contigs) with 99.7% completeness, 0.32% contamination, and no strain heterogeneity. De novo assembly of SILVA database mapped reads resulted in two 16S rRNA gene contigs. Contig 1 (842 bp) showed 100% nucleotide sequence identity (e-value 0.00, bitscore 1350) with the 16S rRNA gene of *Methylosinus sporium* strain NCIMB 11126 as ordered from the DSMZ culture collection. Contig 2 (1431 bp) showed 98.5% nucleotide sequence identity (e-value 0.00, bitscore 2328) to the 16S rRNA gene of *Methylocystis rosea* strain SV97.

## Discussion

Here, we present a proof of concept for the co-cultivation of anaerobic methanogenic archaea and aerobic methanotrophic bacteria in an oxygen-limited bioreactor. Only few studies have previously investigated co-cultivation of methanogens and methanotrophs (Gerritse and Gottschal 1993; Shen et al. 1996; Miguez et al. 1999). These studies showed that co-cultivation of methanogens and methanotrophs is possible, but they did not provide in-depth data on species interactions and system operation. With the present study, we provide a method to study interspecies interactions between methane cycle microorganisms under oxygen limitation. FISH micrographs showed a tight spatial organization between the methanogenic sarcina clusters and layers of aerobic methanotrophic cells. This confirmed our hypothesis that methanotrophs profited from the production of methane from acetate by the methanogens and were therefore closely associated with the methanogens (Fig. 5). Methanogens are most likely protected against oxygen by methanotrophs (Fig. 5).

Genome sequencing of the methanotrophic culture indicated presence of both a *Methylosinus* and a *Methylocystis* species. Both species are members of the alphaproteobacterial family *Methylocystaceae* and possess *pmoA* genes encoding the particulate methane monooxygenase that oxidizes methane at atmospheric levels (Dunfield et al. 1999; Kravchenko et al. 2010). *M. sporium* is a suitable candidate for the co-cultivation set-up due to its high substrate affinity (Murrell et al. 2000). In addition, for *M. trichosporium*, that is closely related to *M. sporium*, *K*_*m*_ values for methane were as low as 0.8–2.0 μM depending on the strain (Joergensen and Degn 1983). Furthermore, *M. sporium* has often been used in previous methanogenic-methanotrophic cultures (Shen et al. 1996; Miguez et al. 1999). Acetate measurements (Fig. 4) showed acetate consumption by the methanotrophic culture. Genome data confirmed the presence of genes encoding acetate kinase (AckA), acetate-CoA ligase (ACSS), and phosphate acetyltransferase (Pta) in both genome bins. It is known that methanotrophs can metabolize acetate, but to our knowledge, growth on acetate and other carbon-carbon bond substrates had been thought to be limited to *Methylocella* and *Methylocapsa* species (Dedysh et al. 2005; Dunfield et al. 2010). The carbon fixation pathways showed the alphaproteobacterial type II pathway of formaldehyde conversion to L-Serine via 5,10-methylenetetrahydromethanopterin (5,10-methylene THMPT).

Co-culture studies could significantly contribute to our current knowledge on methanogen-methanotroph interactions in the environment. The metabolic processes that drive ecosystem-scale GHG fluxes are dependent on the activity of both the aerobic and anaerobic microbial community members (McCalley et al. 2014). Especially the interplay between methanogens and aerobic methanotrophs is relevant, since this determines the types and quantities of GHG fluxes into the atmosphere. The use of pre-defined methanogen-methanotroph co-cultures enables the study of environmental effectors including temperature, pH, substrate, and oxygen availability on methane fluxes and methanogen-methanotroph interactions under controlled conditions. These studies could provide experimental evidence to better estimate wetland GHG fluxes. Methanogen-methanotroph interactions in the environment have recently gained more attention with the contradictory observations of oxic water column methanogenesis and methanogenesis in oxic soils (Bogard et al. 2014; Angle et al. 2017), aerobic methanotrophic activity in anoxic lake waters and sediments (Oswald et al. 2016; Martinez-Cruz et al. 2017), and fermentative activity of gammaproteobacterial methanotrophs under oxygen limitation (Kits et al. 2015). Our study provides a new method to study interspecies interactions of methane cycle microorganisms under an array of environmental conditions.

## Electronic supplementary material

ESM 1 (PDF 147 kb)
