# Supplementary material for: Co-cultivation of the strictly anaerobic methanogen Methanosarcina barkeri with aerobic methanotrophs in an oxygen-limited membrane bioreactor
Source: Appl Microbiol Biotechnol. 2018 May 3;102(13):5685–94. doi: 10.1007/s00253-018-9038-x (PMC5999129; doi:10.1007/s00253-018-9038-x)
Supplement: Supplementary file 1 — (PDF 147 kb) [file 253_2018_9038_MOESM1_ESM.pdf]

Co-cultivation of the strictly anaerobic methanogen *Methanosarcina barkeri* with aerobic methanotrophs in an oxygen-limited membrane bioreactor

Journal: Applied Microbiology and Biotechnology

Michiel H. in 't Zandt[ab], Tijs J.M. van den Bosch[a], Ruud Rijkers[a], Maartje A.H.J. van Kessel[a], Mike S.M. Jetten[abc], Cornelia U. Welte[ac]*

[a] Department of Microbiology, Institute for Water and Wetland Research, Radboud University, Heyendaalseweg 135, 6525 AJ Nijmegen, The Netherlands

[b] Netherlands Earth Systems Science Center, Utrecht University, Heidelberglaan 2, 3584 CS Utrecht, The Netherlands

[c] Soehngen Institute of Anaerobic Microbiology, Radboud University, Heyendaalseweg 135, 6525 AJ Nijmegen, The Netherlands

* Address correspondence to Cornelia U. Welte, c.welte@science.ru.nl

Telephone: +31 24 3652952

Supplementary Information

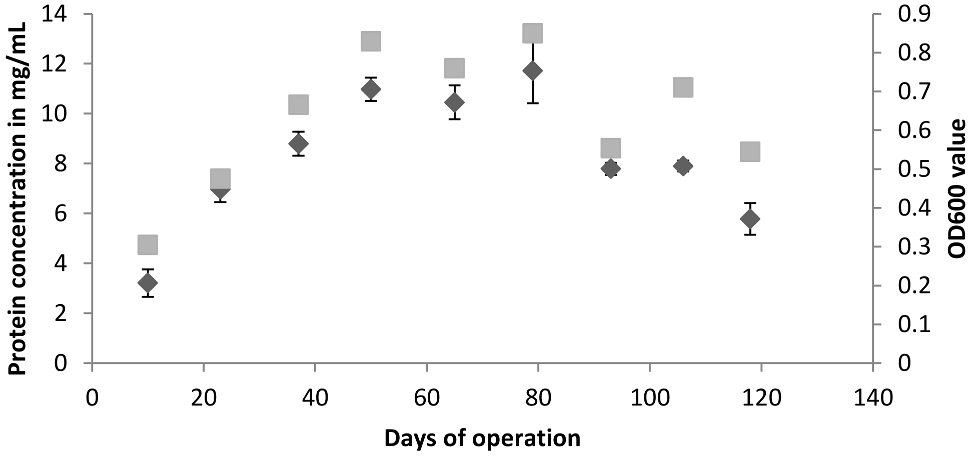

Supplementary Information Figure S1. Combined protein concentration and OD600 analysis of reactor liquid. Diamonds show protein concentrations in mg per mL reactor liquid. Squares show OD600 values for the same samples. n=2. Error bars show standard deviation of the mean.

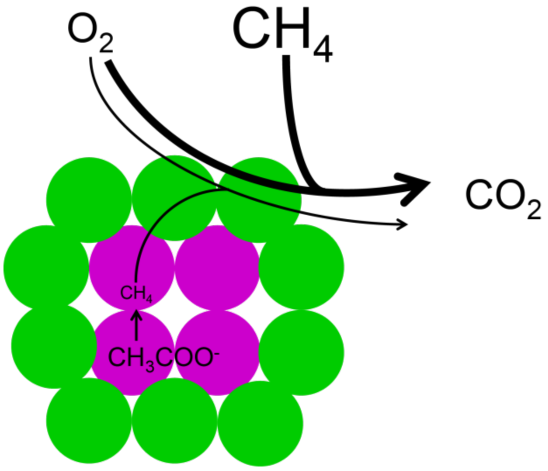

Supplementary Information Figure S2. Hypothesized interaction of aerobic methanotrophs (green) and methanogens (magenta) as observed with FISH microscopy.
